# Trends in Malaria in Odisha, India—An Analysis of the 2003–2013 Time-Series Data from the National Vector Borne Disease Control Program

**DOI:** 10.1371/journal.pone.0149126

**Published:** 2016-02-11

**Authors:** Ashirbad Pradhan, Anita Anasuya, Madan Mohan Pradhan, Kavitha AK, Priyanka Kar, Krushna Chandra Sahoo, Pinaki Panigrahi, Ambarish Dutta

**Affiliations:** 1 Centre for Disease Epidemiology and Surveillance, Asian Institute of Public Health, Bhubaneswar, Odisha, India; 2 Department for International Development, United Kingdom supported Technical and Management Support Team, Bhubaneswar, Odisha, India; 3 National Vector Borne Disease Control Programme, Department of Health and Family Welfare, Government of Odisha, Bhubaneswar, Odisha, India; 4 Center for Global Health and Development, College of Public Health, University of Nebraska Medical Center, Omaha, United States of America; 5 Indian Institute of Public Health, Public Health Foundation of India, Bhubaneswar, Odisha, India; Centro de Pesquisa Rene Rachou/Fundação Oswaldo Cruz (Fiocruz-Minas), BRAZIL

## Abstract

**Background:**

Although Odisha is the largest contributor to the malaria burden in India, no systematic study has examined its malaria trends. Hence, the spatio-temporal trends in malaria in Odisha were assessed against the backdrop of the various anti-malaria strategies implemented in the state.

**Methods:**

Using the district-wise malaria incidence and blood examination data (2003–2013) from the National Vector Borne Disease Control Program, blood examination-adjusted time-trends in malaria incidence were estimated and predicted for 2003–2013 and 2014–2016, respectively. An interrupted time series analysis using segmented regression was conducted to compare the disease trends between the pre (2003–2007) and post-intensification (2009–2013) periods. Key-informant interviews of state stakeholders were used to collect the information on the various anti-malaria strategies adopted in the state.

**Results:**

The state annual malaria incidence declined from 10.82/1000 to 5.28/1000 during 2003–2013 (adjusted annual decline: -0.54/1000, 95% CI: -0.78 to -0.30). However, the annual blood examination rate remained almost unchanged from 11.25% to 11.77%. The keyinformants revealed that intensification of anti-malaria activities in 2008 led to a more rapid decline in malaria incidence during 2009–2013 as compared to that in 2003–2007 [adjusted decline: -0.83 (-1.30 to -0.37) and -0.27 (-0.41 to -0.13), respectively]. There was a significant difference in the two temporal slopes, i.e., -0.054 (-0.10 to -0.002, p = 0.04) per 1000 population per month, between these two periods, indicating almost a 200% greater decline in the post-intensification period. Although, the seven southern high-burden districts registered the highest decline, they continued to remain in that zone, thereby, making the achievement of malaria elimination (incidence <1/1000) unlikely by 2017.

**Conclusion:**

The anti-malaria strategies in Odisha, especially their intensification since 2008, have helped improve its malaria situation in recent years. These successful measures need to be sustained and perhaps intensified further for eliminating malaria from Odisha.

## Introduction

Being a major contributor to human morbidity, mortality, and economic adversity, malaria is a significant public health problem in India[1,2]. With a ubiquitous year-round presence in the country, it exacts an unacceptable toll on the health of people of all ages[3]. As per the World Malaria Report (WMR) 2013, India contributed to about 52% of the two million confirmed malaria cases in South-East Asia.

India’s varied geography, ecological diversity, and climatic variability make it an ideal place for the widely-spread mosquito vectors to breed and transmit malaria parasites[4], albeit with varied intensity in different parts of the country[5]. The north-eastern, central, and eastern states of India are regarded as high malaria transmission zones accounting for nearly 80% of the total malaria incidence and deaths reported in the country[6,7]. Among these, the worst affected state is Odisha[8].

Odisha, with only about 4% land area and 3% population of India, accounted for 26.9% of India’s malaria cases, and about 17.6% of all reported deaths in 2013[9]. Of its 30 districts, the bulk of the incidence is reported from the Southern, Western and Northern belt whichare mostly covered with forest and hilly areas. These ecological features along with its tropical climate characterized by high temperature, high humidity and medium to high rainfall provide the most favourable and conducive environment for breeding of vectors and development of malaria parasites, thus making it highly vulnerable to malaria[10].

Given the gravity of the problem, malaria control has always been one of the top health priorities for the state. Since the inception of the National Malaria ControlProgram(NMCP) in the 1950s in the country, the state has adopted various malaria containment measures, mainly in convergence with the national anti-malaria strategies. Over the years, the state’s malaria control effort has witnessed many transformations coinciding with the periodic changes in national malaria control strategiesfrom NMCP to National Malaria Eradication Program (NMEP), and then National Anti-Malaria Program (NAMP) and presently National Vector Borne Disease Control Program (NVBDCP). The malaria control program in the state received a major boost in the later part of the 1990s with the introduction of new strategies in the national malaria agenda, often through various externally-aided projects like Enhanced Malaria Control Project(EMCP) in 1997 and Intensified Malaria Control Program (IMCP) in 2005, which were supported by the World Bank and The Global Fund to Fight AIDS, TB and Malaria (GFATM), respectively. The Department for International Development (DFID), United Kingdom (UK) supported Odisha Health Sector Plan (OHSP), 2007 is another such initiative in the direction of state’s malaria control efforts. The main components of these intensified anti-malaria strategies included integrated vector control measures, use of newer techniques for early diagnosis and effective treatment, service decentralization, behavior change communication (BCC), improved surveillance, and inter-sectorial convergence. Since 2002, these strategies are being implemented in the state under the NVBDCP [11–13].

Recent reports from the NVBDCP suggest that the malaria situation in Odisha has improved considerably over the years, notwithstanding the various pitfalls in the anti-malaria activities mounted by the public health system of the country. However, the malaria trends in Odisha have hardly been studied. Hence, this study aimed to present the evolving spatio-temporal profile of malaria in the state during 2003–2013, in light of the various anti-malaria measures undertaken. It also aimed to predict the future trends related to the disease, so as to inform anti-malaria policy. Additionally, this study evaluated the disease trend in the state, before and after the roll-out and scale-up of a package of interventions against malaria, implemented in 2008, to detect whether there was any significant effect of such intensified activities, greater than the secular trend.

## Materials and Methods

This is a mixed methods study with a convergent design. The quantitative programmatic data were used to compare the malaria occurrence trends before and after a package of intervention was rolled-out or scaled-up in 2008, and the qualitative data were simultaneously collected to identify the timing and characterise the implemented intervention packages.

### Time-series data

District-wise (30 districts) monthly malaria surveillance data (2003–2013) obtained from the NVBDCP, Odisha, were converted into the annualized rates of malaria incidence and blood examination.The blood examination rate,an important indicator of the surveillance efficacy of the malaria program,signifies the blood slides examined per 100 populations, collected from the individuals suffering from fever presumed to be due to malaria [14].After that, these two rates were structured as multiple month-wise time-series. The mid-year district-wise population was used for computing these rates derived from the national censuses (2001 and 2011) and for accounting for the growth of the population. Blood examination included both microscopy-based blood slide examination and blood samples examined by Rapid Diagnostic Tests (RDTs). Data entry and analysis were conducted using the Epi Info v.7.1.4 and R v3.0.1 software, respectively[15].

### Qualitative information

Following informed consent, we conducted sixteen semi-structured key-informant interviews on anti-malaria strategies adopted by the state during 2003–2013 with stakeholders from the NVBDCP, Odisha, and OHSP to explore the trends in detail.

### Ethics statement

The data used in our analysis were district-aggregate programmatic data of NVBDCP, collected in de-identified form from the Odisha bureau of the program. These were routinely collected by the program information system during 2003 through 2013 before the analysis was undertaken in 2014. No individual data were analyzed for our study. Hence there was no scope or need for anonymization or de-identification of the data. Thus, the ethical consideration was deemed to be inapplicable to the quantitative data. However, for the stakeholders’ interview, approval was obtained from the Institutional Review Board of Asian Institute of Public Health, Odisha, India. The ethical review board suggested that for secondary programmatic data, approval from the concerned department was enough. Hence, prior departmental permission from the NVBDCP, Odisha was taken. The informed and written consents were taken from all the participants before the interview.

### Data analysis

#### Estimation of trend and seasonality

The time-series data were decomposed to describe their seasonality and secular trends. The trends were graphically examined using raw time-series data, centered moving averages, locally weighted, and linear regression lines. The decomposed seasonality was further explored to determine monthly fluctuations from the mean.

The unadjusted annual trends were initially estimated using generalized least square regression[16,17] models, including time as the only explanatory variable. A correlogram and partial correlogram of the residuals were graphically plottedat each time point as the lag. The underlying structures of the serial dependence of the residuals were examined, and their order of Auto Regression and Moving Averages parameters were estimated. These parameters were then included in the next models to control for the serial dependence of the residuals. A multivariable model adjusted for timetrends in malaria incidence was then estimated after controlling for blood examination rates.

#### Analysis of the pre and post-intensification periods

Significant strategic changes in the national and state anti-malaria programs and roll-out and scaling-up of those new anti-malaria interventions, along with the strengthening of some of the ongoing strategies occurred in the state during 2008–2009. Hence, we analyzed the malaria time-series data after dividing them into two periods i.e. interrupting the time series into two segments: the first segment ranging from 2003 to 2007, denoting the pre-intensification period, and the second segment ranging from 2009 to 2013, denoting the post-intensification period. The year 2008hereinafter referred to as the period of “intensification”, was excluded from this analysis of both pre and post-intensification segments as this was the year considered for ramping up the activities. The slopes of the time-trends of malaria incidence between these two segments were compared using a segmented regression model of the interrupted time-series[18–20], which estimated the quantum of change in the slopes of these two segments and also tested the statistical significance of that change. We also conducted a sensitivity analysis using the same methodology, after including the year 2008 in the pre-intensification period[18].To confirm the robustness of the segmented regression model and identify the optimum timepoint at which the significant change(s) occurred after the intensification of anti-malaria activities, we conducted a change-point analysis[21], by using the R change-point package that employs the Hinkley algorithm.

#### Analysis of the districts stratified by the baseline disease burden

Districts were stratified as per their annual malaria incidencein 2003 (S1 Table). The districts with an annual blood examination rate of <10% were corrected as per the NVBDCP guidelines and then stratified into the following four clusters: 0–1.9 = “Low,” 2–4.9 = “Moderate,” 5–9.9 = “High” and ≥10 = “Very High” (VH), the numbers denoting the annual malaria incidence in 2003. Then the trends in malaria incidence in these four clusters were estimated using the same process as described above.

#### Prediction

The malaria incidence was predicted for the next three years (2014–2016) using historical disease incidence rates (2003–2013) and the Holt-Winters exponential smoothing forecasting models. The appropriateness of the predictive model was checked using the Box-Ljung test.

#### Spatial distribution of malaria

The districts stratified as per the aforesaid cut-off points were color-coded in the statemap once at baseline (2003) and then for 2008 and 2013, to display the progress of each district with regards to their disease burden.

#### Analysis of the qualitative data

The information from the key-informants was noted during the interview by one researcher while the other conducted the interview. After each interview, the researcher debriefed the data and adopted a new guide based on the previous findings. The qualitative data were analyzed using content analysis[22]. Meaning units were identified and coded. Similar codes were clustered together and merged into categories. The main theme- emergent concepts were developed based on the interpretation of codes and categories.

## Results

During the eleven years (2003–2013), the annual malaria incidence of Odisha decreased from 10.82 per 1000 population in 2003 to 5.28 in 2013, whereas the annual blood examination rate remained almost unchanged from 11.25% in 2003 to 11.77% in 2013 (Table 1).

**Table 1:** Annual Malaria Incidence and Blood Examination Rate, 2003–2013, Odisha.

Year	Malaria cases detected by the NVBDCP	Blood slides examined by the NVBDCP	Population	Annual Malaria Incidence per 1000 population	Annual Blood Examination Rate (%)
**2003**	409445	4256451	37833200	10.82	11.25
**2004**	398305	4188029	38347469	10.39	10.92
**2005**	391830	4770794	38861739	10.08	12.28
**2006**	376214	4920147	39376009	9.55	12.50
**2007**	364318	4805306	39890279	9.13	12.05
**2008**	343778	4790798	40404549	8.51	11.86
**2009**	359493	4826635	40918818	8.79	11.80
**2010**	364432	4971009	41433088	8.80	12.00
**2011**	308374	4659729	41947358	7.35	11.11
**2012**	248948	4555739	42633052	5.84	10.69
**2013**	227990	5078508	43147321	5.28	11.77

### Trends in malaria in Odisha, 2003–2013

**Fig 1:**
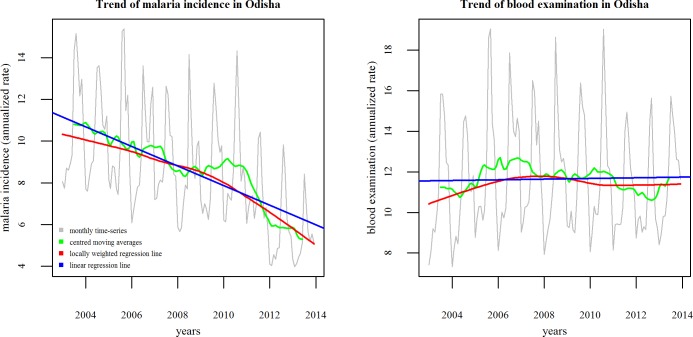
Trends in malaria incidence and blood examination from 2003–2013, Odisha.

There was a significant annual decline in the malaria incidence in the state, the linear trend being -0.49 per 1000 population (95% CI: -0.60 to -0.37, p<0.0001), which increased to -0.54 (-0.78 to -0.30, p<0.0001) after adjustment for blood examination. In contrast, the blood examination rate underwent hardly any change with a change of -0.02 percentage points (-0.12 to 0.08, p = 0.705) over the same period (Fig 1).

#### Seasonal variability

The highest peak of this seasonal disease in almost all the years was observed during July–August, showing a 41% increase in the incidence as compared to the annual mean. The minimum malaria incidence was observed during January, which was 25% less than the meanannual incidence (S1 Fig). However, since 2008, the peak incidence of the disease showed a slight shift towards August.

### Trends during the pre-intensification vs. post-intensification periods (2003–2007 vs. 2009–2013)

**Fig 2:**
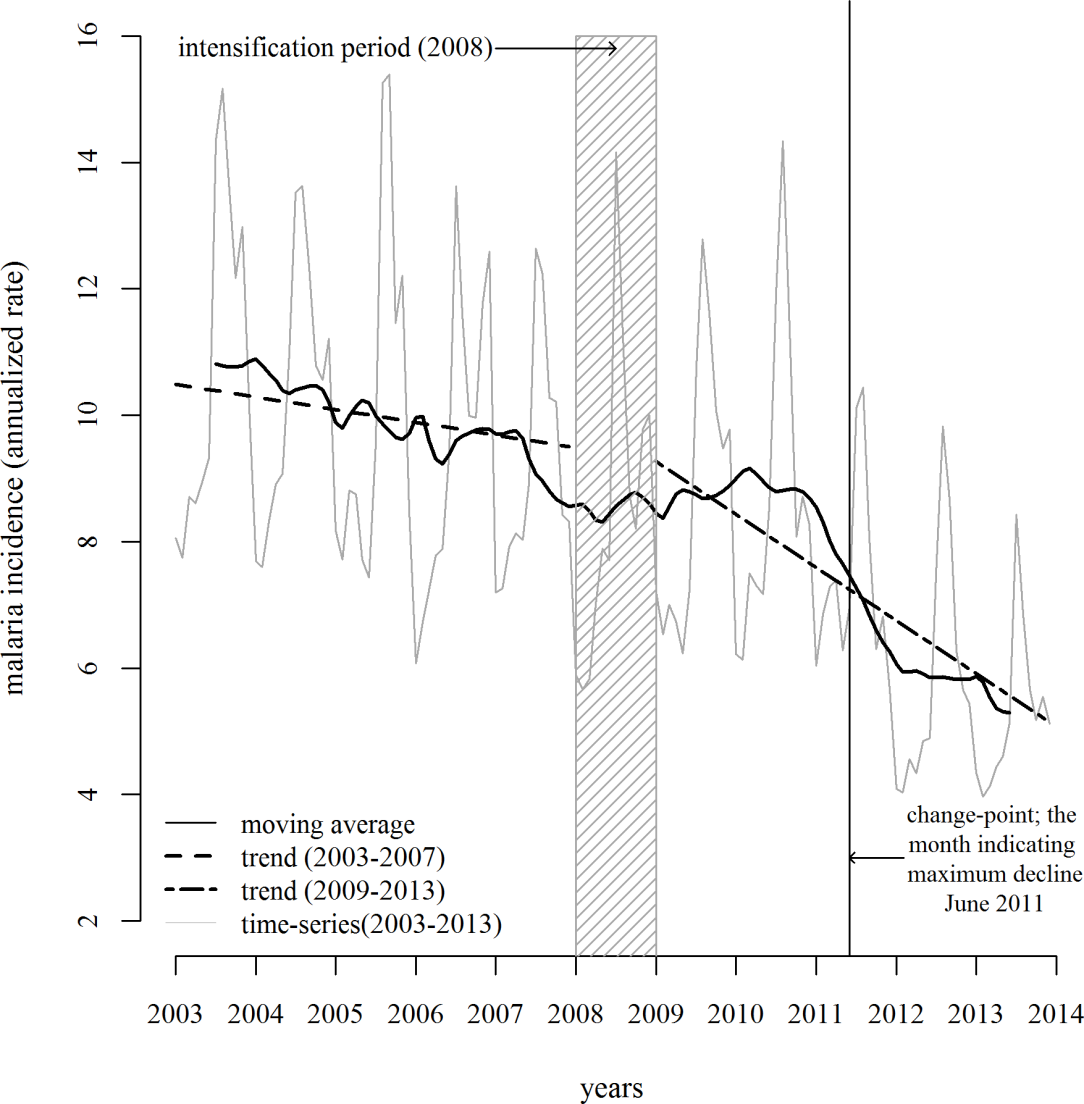
Malaria incidence during 2003–2007 and 2009–2013, Odisha.

The overall linear slope of decline in the malaria incidence between 2003 and 2007 was considerably flatter than that for 2009–2013 (Fig 2).

The estimated annual decline for 2003–2007 was -0.19 per 1000 population (-0.85 to 0.46, p = 0.56), which increased to -0.27 per 1000 population (-0.41 to -0.13,p<0.0001)after adjustment for blood slide examination. In comparison, the annual decline during 2009–2013 was -0.81 (-1.46 to -0.18,p = 0.015), which slightly increased to -0.83 (-1.30 to -0.37, p<0.0001) after adjustment (Table 2). Further, the blood examination rate in the state increased by 0.34 percentage points (0.12 to 0.56, p = 0.013) annually during 2003–2008, whereas the change during 2009–2013 was not significant.

**Table 2:** Trends in Malaria Incidence, 2003–2013, Odisha.

	Unadjusted trend estimate* of malaria incidence	Trend estimate* of malaria incidence adjusted for blood examination rate
**Odisha, 2003–2013**	-0.49 (-0.60 to -0.37), p<0.0001	-0.54 (-0.78 to -0.30), p<0.0001
**Two periods**		
Odisha, 2003–2007	-0.19 (-0.85 to 0.46), p = 0.56	-0.27 (-0.41 to -0.13), p<0.0001
Odisha, 2009–2013	-0.81 (-1.46 to -0.18), p = 0.015	-0.83 (-1.30 to -0.37), p<0.0001
**Strata as per disease burden at baseline, 2003**		
Very High	-0.78 (-1.06 to -0.49), p<0.0001	-0.69 (-0.94 to -0.33), p<0.0001
High	-0.34 (-0.44 to -0.25), p<0.0001	-0.44 (-0.48 to -0.40), p<0.0001
Moderate	-0.24 (-0.29 to -0.19), p<0.0001	-0.27 (-0.31 to -0.23), p<0.0001
Low	-0.04 (-0.08 to -0.01), p = 0.013	-0.04 (-0.08 to 0.00), p = 0.06

*Trend estimates are per 1000 population per year.

The segmented regression of the interrupted time-series demonstrated a significant difference in the temporal slopes between the pre-intensification (2003–2007) and post-intensification (2009–2013) periods, separated by the intensification phase of 2008. The difference in the two slopes was -0.054 (-0.10 to -0.002. p = 0.04) per 1000 population per month, indicating almost a 200% greater decline in the post-intensification period as compared to that in the pre-intensification period (Fig 2). The sensitivity analysis with the inclusion of 2008 in the pre-post analysis only changed the results marginally.

The intensification phase of 2008 was followed by a surge in case detection between 2009 and 2010 (Fig 1). This was followed by a steep decline from 2011, the sharp decline somewhat slowing down in 2012, as evident from the linear smoothers in Fig 2. The change-point analysis showed that the maximum change during 2003–2013 occurred in the month number 102 in the time-series, which was June 2011. During that month, the maximum decline was experienced, as also evident from the visual exploration of the time-series (Fig 2).

### Trends in the districts stratified by the baseline disease burden

**Fig 3:**
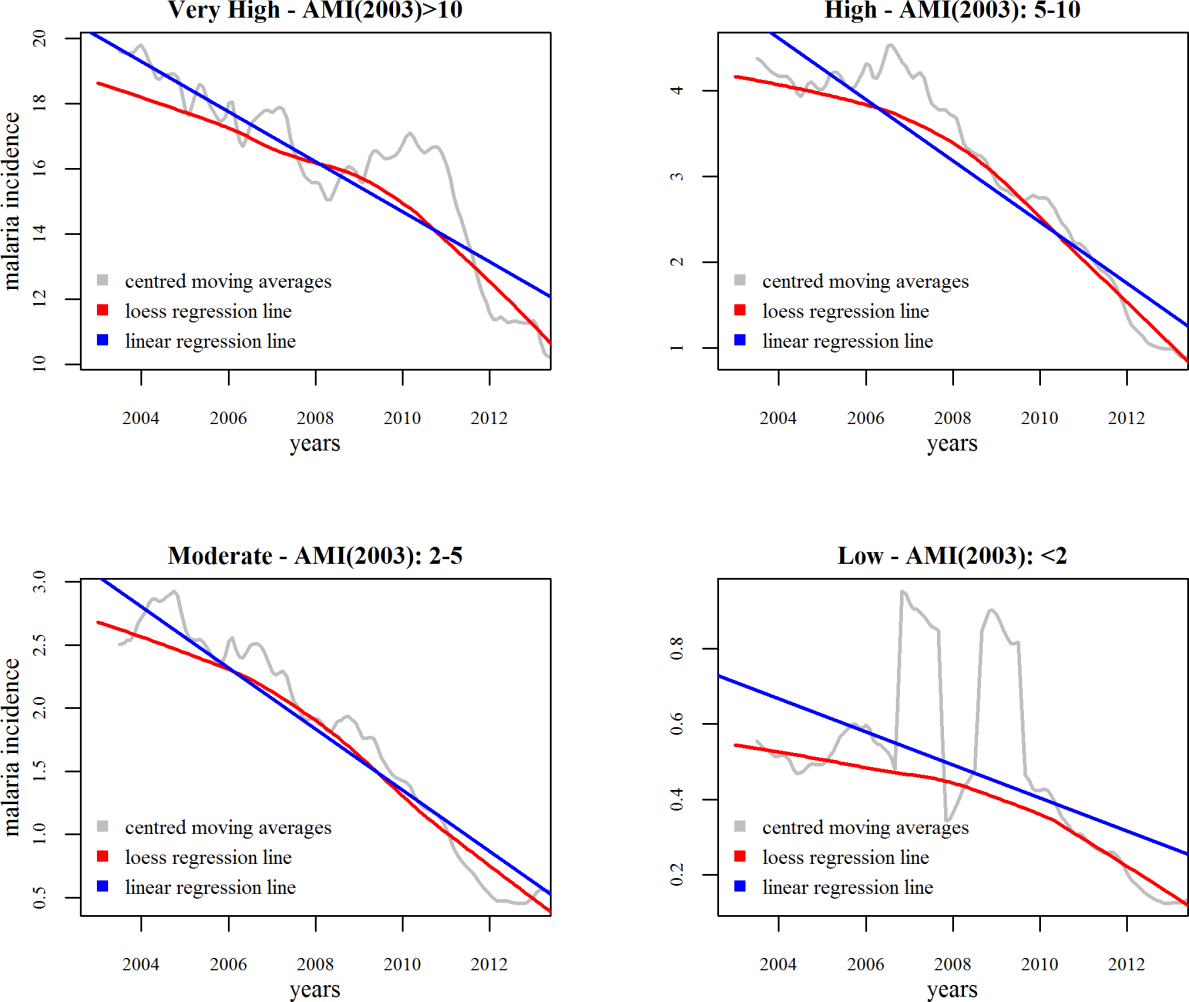
Trends in malaria in the districts stratified by their baseline malaria burden (2003), Odisha.

The annual relative decline in the malaria incidence in Low burden cluster of districts had a lesser gradient than did the other three clusters of districts, where the declines were comparable (Fig 3).The blood examination rate-adjusted gradients were -0.69 (-0.94 to -0.33, p<0.0001), -0.44 (-0.48 to -0.40, p<0.0001), -0.27 (-0.31 to -0.23, p<0.0001), and -0.04 (-0.08 to 0.00, p = 0.06) for the VH, High, Moderate and Low clusters, respectively. For the first three clusters, the annual decline, although different in absolute terms, was very similar regardingthe decline relative to their baseline.

The blood examination rate registered a decline in the VH cluster over the years, with the annual percentage point decline estimated to be -0.29 (-0.45 to -0.14, p<0.0001). The other three clusters experienced increase in the blood examination rates during these eleven years, the annual estimates being 0.20 (0.009 to 0.39, p = 0.042), 0.18 (0.05 to 0.30, p = 0.005), and 0.28 (0.24 to 0.34, p<0.0001) forthe High, Moderate and Low clusters, respectively.

### Prediction

**Fig 4:**
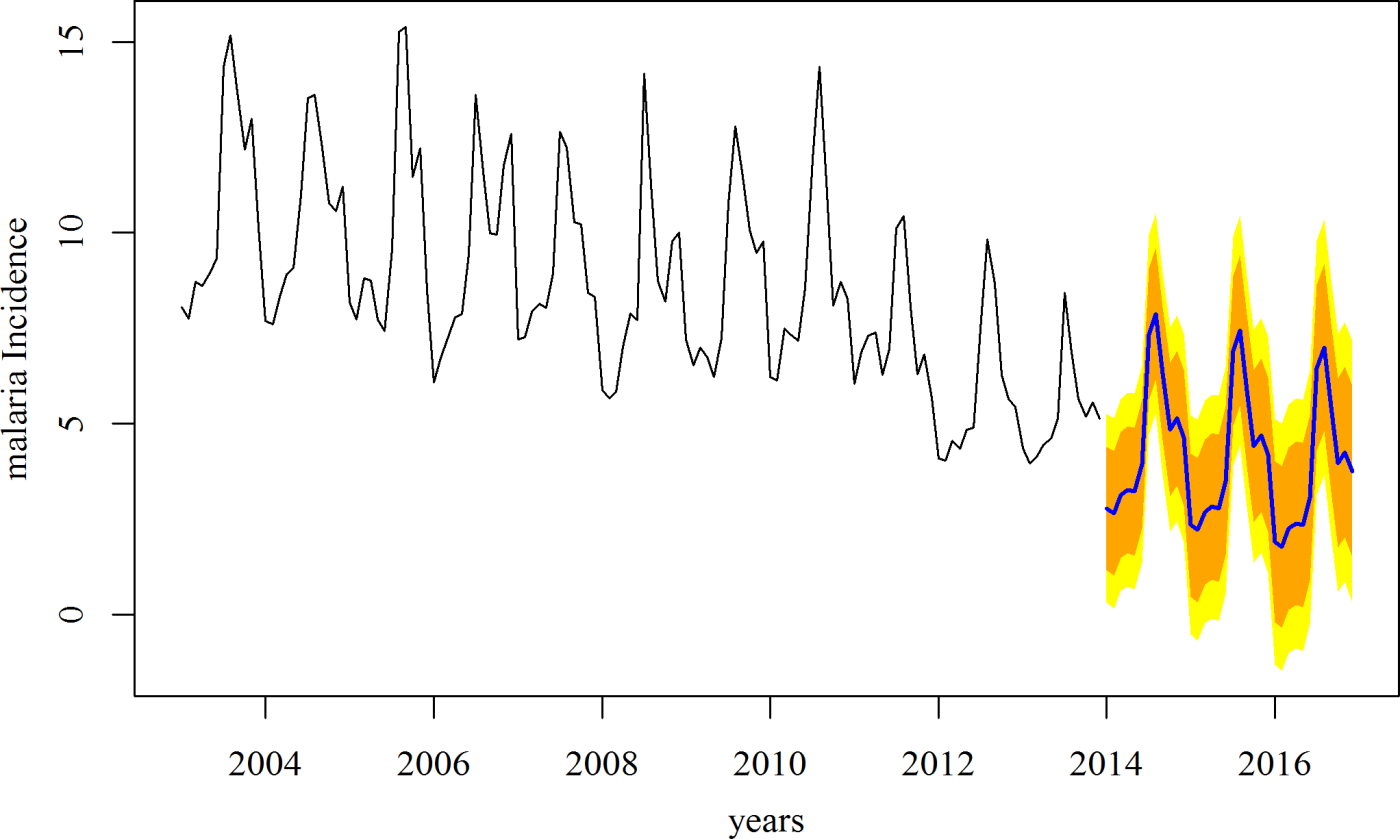
Forecasting malaria incidence for 2014–2016 based on the trends from 2003–2013, Odisha.

The forecast for the next three years (2014–2016) (Fig 4) showed that as compared to the rapid decline of 2011–2012, the decline was likely to slow down. This implies that the elimination level of annual malaria incidence of ≤1/1000 might not be achieved by 2017 for the whole state, which is the current goal of the NVBDCP. The districts in the Low and Moderate incidence clusters had already reached this elimination level, and the High incidence cluster is quite likely to reach it by 2017 (Fig 2). The VH incidence cluster started out at very highlevels and showed the steepest decline over the eleven-year period. However, as per the current state, it is unlikely to reach the elimination level by 2017.

### Spatial distribution of malaria

**Fig 5:**
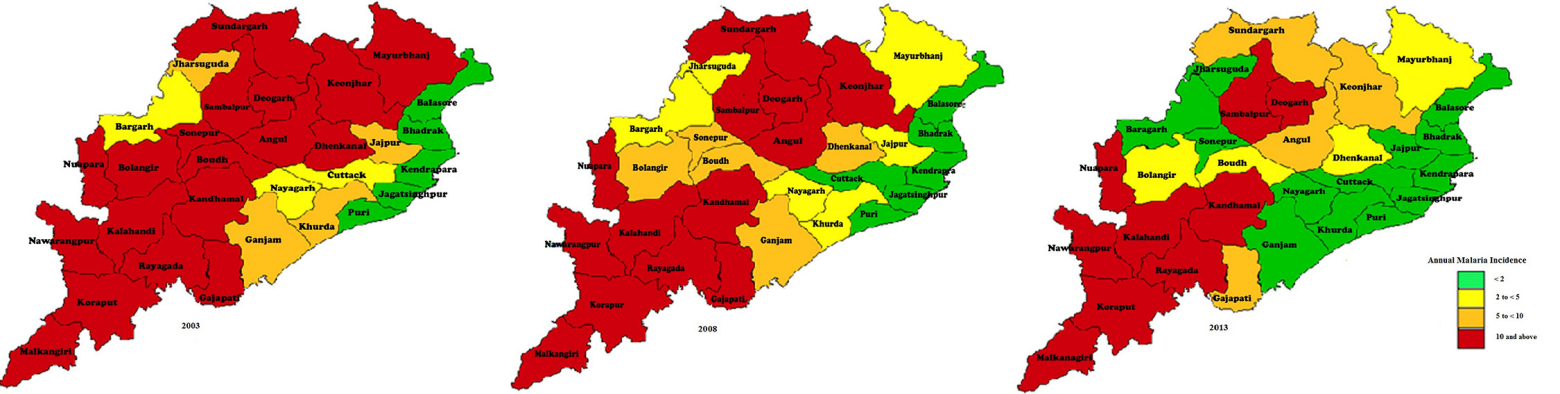
Annual malaria incidence (2003, 2008, and 2013), Odisha.

Malaria continued to ravage the seven southern districts of Kandhamal, Kalahandi, Rayagada, Koraput, Nawarangpur, Nuapada, and Malkangiri, and two central districts of Sambalpur and Deogarh, which were consistently in the VH incidence zone from 2003 through 2013.More apparent success had been achieved in north-western districts, some of which have moved from the VH to other less-burden clusters. The five coastal districts remained in the Low cluster throughout the study period (Fig 5).

### Increased administrative and political commitment to reduce the malaria burden in the state

The information from key-informants revolved around various anti-malaria measures undertaken during 2008–2013 vis-à-vis that of 2003–2007. As per the perception of the informants, during 2003–2007, no new strategy other than the routinely implemented ones were used against the malaria challenge in the state, leading to the deceleration of the consolidation of the success achieved in the early nineties. Interviewees particularly revealed a massive roll-out of artemisinin-based combination therapy (ACT) and RDT replacing and supplementing microscopy-based blood examination by the NVBDCP in the state, especially in the VH districts during 2008. These steps were implemented in compliance with the Revised National Drug Policy, 2007. They also stated that this period also experienced a considerable surge in other existing and new activities, which were sustainedthereafter. The main theme that emerged from the key informant interviews was “Increased political and administrative commitment to reduce the malaria burden in the state” since 2008,underlying the “intensification” comprising of a massive roll-out of new and scale-up of existing intervention strategies, which evolved from the categories explained in Table 3. The interviewees also perceived that the surge in the programmatic inputs had been instrumental in improving the malaria situation in the state.

**Table 3:** Anti-malaria Inputs Rolled out from 2008–2013, Odisha.

Theme	Increased administrative and political commitments to reduce the malaria burden in the state				
**Categories**	Vector control	Case detection and management	Behavioral Change Communication (BCC) strategies	Human resources	Supportive measures
**Codes**	Free distribution of Long Lasting Insecticidal Nets(LLINs) in high endemic districts	Set up of fever treatment depots (FTD) at the community level	Innovative BCC campaigns promoting the use and maintenance of bed-nets, e.g.,“Nidhi Mousa To Masari Ne”^b^	A multi-disciplinary strong technical team at the National Vector Borne Disease Control Program (NVBDCP)	Highly supportive bureaucratic and administrative environment
	Successful implementation of the “Mo Masari scheme”^a^	Sufficient supply and wider coverage of Rapid Diagnostic Kits (RDKs) and artemisinin-based combination therapy (ACTs)	Social mobilization drives through the “Nidhi Ratha”^c^ and folk theater resulting in improvement in treatment seeking behavior	Deployment of Accredited Social Health Activists (ASHAs) in anti-malaria activities	Regular fixed day technical committee meetings
	Wider Indoor Residual Spray (IRS) coverage		Health messages transmission through interpersonal communication by frontline health workers	Capacity building of ASHAs through training on malaria diagnosis using Rapid Diagnostic Tests (RDTs), and anti-malaria drug administration	Multiple stakeholders’ involvements
				Provision of malaria technical supervisors, vector-borne disease consultants, and trained health workers for malaria	Financial and technical support from the DFID, World Bank, GFATM, and World Health Organization
					Government willingness on extensive investment on LLINs
					Strengthening of malaria surveillance and information systems by using standardized formats

a“Mo Masari” or “my mosquito net” is an endeavor by the Government of Odisha to protect all pregnant mothers, and under five and tribal school children in highly endemic areas

b A pre-publicity BCC campaign to generate demand for mosquito nets and demonstration of their use during LLINs distribution

c“Nidhi Ratha” the name of a chariot used as a part of the “Nidhi Mousa To Masari Ne” campaign for imparting messages on malaria prevention and control; and usage of LLIN by organizing folk theatres in Odia language and distributing leaflets throughout its journey.

## Discussion

The present study examined the temporal trend in malaria morbidity in Odisha from 2003–2013, with the state achieving a 44.32% reduction in confirmed malaria infections (an average decline of approximately 5% every year from its annual incidence in 2003, when it was almost eleven per 1000 population). The maintenance of a steady blood examination rate in the state during this period, a critical indicator of effective malaria surveillance[2], was noticeable during this entire period. However, the average annual decline in the incidence during 2009–2013 was three times steeper than that during 2003–2007, notwithstanding the peak in malaria case detection in 2009–2010. This considerable difference in temporal slopes of malaria incidence between the pre-intensification and post-intensification periods has a likely association with intensification of anti-malaria activities in 2008, as our study also found large-scale introduction of new and reinforcement of existing intervention packages in the state, beginning in 2008, driven by a noticeable surge in political and administrative commitment in the fight against malaria in Odisha.

The ensuing peak in 2009–2010, following intensification, perhaps signifies the increase in case detection with the massive surge in anti-malaria activities preceding this period. It is likely that the efficient management of a vast number of detected cases within a short time-period using ACT led to a rapid contraction of the reservoir of malaria parasites in the community. This, in coordination with the other efforts undertaken during this period of intensification, perhaps ushered in the sharp decline in the malaria incidence from 2011, the timing of which was also formally endorsed by the change-point analysis of our data. This sharp decline accounted for the steeper overall decline observed during the post-intensification period as compared to the slower decline in the pre-intensification period. Our study underscores, perhaps for the first time in the Indian context, the association between the intensification of anti-malaria measures and asignificantly greater decline in malaria occurrence in the post-intensification era. The strengthening of the existing measures and implementation of new measures gained momentum from 2008. This was afterseveral majortechnical changeswere introducedin the malaria drug policy(2007), the implementation of which werestrongly supported by increased political and administrative commitment from the state policy-makers and anti-malaria service delivery system of Odisha, as revealed by the key informants from various levels of the NVBDCP in the state during their interviews. Other studies conducted in different countries such as Kenya [23], Rwanda[24], Zambia[25], and Zanzibar [26] also provide similar evidence that the decline in malaria incidence coincides with the scale-up of thecurrent malaria control and preventive measures along with the strong political will.

Further, to validate the information on the intensification of the anti-malaria activities during 2008–2009, we explored the recently published Lot Quality Assurance Sampling (LQAS) survey results[27]. The LQAS was used to evaluate the coverage and performance of various programmatic inputs by the NVBDCP for the mid-term course-correction[12]. It showed a remarkable increase in various programmatic components in the initial districts, such as protection of adults and children (27% and 42% increase, respectively) through the use of LLINs, improved treatment-seeking behavior, and increased treatment of diagnosed cases (average increase of 63%). However, Valadez et al. also underscored the weaknesses in the program, such as lack of knowledge regarding maintenance of LLIN, thus compromising their lifespan and occasional stock-outs of RDTs in some districts.

The sharp decline in the malaria incidence from 2011 slowed down in 2012 and 2013, as was also reflected in the prediction for the next three years (2014–2016), thus raising concerns for possible deceleration of the success achieved in the state. This “slowing down” was perhaps due to the non-renewal of the LLINs in the state after the expiry of the shelf-life of the current crop, the majority of which were distributed in 2008[27]. However, the comparative contribution of each individual specific anti-malaria measure to this decline, using district-wise analysis, could not be explored quantitatively because of the absence of robust data owing to reporting inconsistency and incompleteness. Thus, we relied on the qualitative information to suggest that the considerable decline in the malaria incidence in the state was due to the intensification of malaria control interventions. Despite this,the goal of achieving a state-wide malaria incidence of <1/1000 by 2017[12] is unlikely to be met as the seven southern districts continued to be in the VH (>10/1000) zone consistently over the last 11 years, though each of these districts achieved remarkable decline as compared to their higher baseline burdens. The districts belonging to the other clusters either had achieved or are likely to achieve this elimination target by 2017. Sustaining or even heightening the current anti-malaria measures with proven past success as demonstrated in our analysis, with targeted resourceallocation for VH burden districts or blocks, may be the necessary strategy for the “last push” to bring malaria to the elimination level in the whole state. This would offset any trend of slackening in surveillance in those areas, as might be evident by the decline in the blood examination rate in the VH cluster. However, perhaps much of that decline in blood examination could be ascribed to the substantial reduction of fever cases in the community due to the rapid decline in the malaria burden and also the change in the NVBDCP policy on blood examination, whereby many obvious non-malarial causes of fever were excluded[28]. Additionally, this decline in the blood examination rate in this cluster is perhaps a testament to the rapid decrease in malaria fevers in these communities.

There are some limitations to the present study. The first one could be the use of secondary surveillance data of the NVBDCP, which included the cases detected and reported by the program. This may not have been a true reflection of all the malaria cases in the community, as shown by other studies in India[29,30]. Those “left-out” by the program might be seeking care from other sources such as private or non-formal medical systems. Despite this, it could be argued that such an underestimation due to the non-notification from the private sector is unlikely to influence our time-trend estimates substantively because the portion of the “uncovered” population was unlikely to have increased with time. Hence, the decline we notice is very unlikely to be due to decreasing program coverage; rather, is likely to be related to the mounting of effective preventive and curative strategies by the NVBDCP. Further, the pool of “uncovered” malaria cases would have reduced over time as many measures were successfully undertaken to increase the access to the NVBDCP in the state. Moreover, the strength of this study is that the decline of malaria incidence as observed was adjusted for a key operational variable, i.e., blood examination rate, which accounted for fluctuations in program surveillance. The blood examination rate remaining largely unchanged during the period of our analysis, indicates that program surveillance did not undergo any significant decline during the study period. Further, the intensification of anti-malaria activities undertaken in 2008 saw a major overhaul of microscopy-based blood examination strategy, as it was largely replaced by RDT [31]. This bolsteredthe diagnosis of malaria cases, evident from the resulting surge of cases during 2009–2010.Second,the pre-post difference in the slopes could not be compared with a control area that did not receive an intensified package. This can be argued as a limitation of the study, weakening the causal inferences between the intensification package and the post-intensification sharper decline. However, segmented regression of interrupted time-series, as used in our study, is considered a robust method to study such effectiveness of policies and strategies [32,33], even in the absence of a control, though causal inferences have to be carefully drawn.

Other factors such as climatic variables, e.g. rainfall, humidity, and temperature, have an impact on the malaria epidemiology [34,35]. Unfortunately,data regarding these factors were either not available or unavailable before 2006, to be considered in our models. However, it is unlikely that changes in these atmospheric variables over the 11 years considered, have singlehandedly brought about the decline in the malaria situation in Odisha as the rainfall data of Odisha, available in the public domain beyond 2006, does not show much deviation from normal[36]. Moreover, the seasonal peaks and troughs observed in the malaria incidence also corresponded with the rainfall pattern in the state.

Another limitation may be the lack of quantitative data regarding the intensification of the anti-malaria activities in 2008. However, the exploration of the situation using qualitative techniques has pointed towards the intensification of many anti-malaria activities in 2008. Additionally, the intensification of many of such new and ongoing efforts such as political commitment and supervisory efforts are often better explored qualitatively.

## Conclusion

To conclude, our study, perhaps for the first time to our knowledge, has systematically estimated the malaria trends in Odisha and has shed light on the substantial decline in the malaria incidence in the state during the last 11 years (2003–2013).Scaling-up, and rolling out of existing and various new anti-malaria strategies since 2008 in the state was found to be significantly associated with this decline, the bulk of which being achieved between 2009–2013, especially in the districts withthe maximum disease burden,a likely pointer to the effectiveness of these intensification strategies. Nevertheless, many districts continue to have a very high burden of malaria, and hence, a stagnation of the success or resurgence of the problem in those areas and the neighboring regions cannot be ruled out. Therefore, the package ofactivities and control measures that was found to be associated with the success in our studyneeds to be sustained or even increased in future.

## Supporting Information

S1 Fig Average seasonal variability of malaria incidence (2003–2013), Odisha. (TIF)

S1 File Certificate of Professional Scientific Editing. (PDF)

S2 File Revised manuscript with track changes. (DOCX)

S1 Table Status of Annual Malaria Incidence in 2003 (Baseline year), Odisha. (PDF)
