# Supplementary figures and images for: Trends in Malaria in Odisha, India—An Analysis of the 2003–2013 Time-Series Data from the National Vector Borne Disease Control Program
Source: PLoS One. 2016 Feb 11;11(2):e0149126. doi: 10.1371/journal.pone.0149126 (PMC4750863; doi:10.1371/journal.pone.0149126)

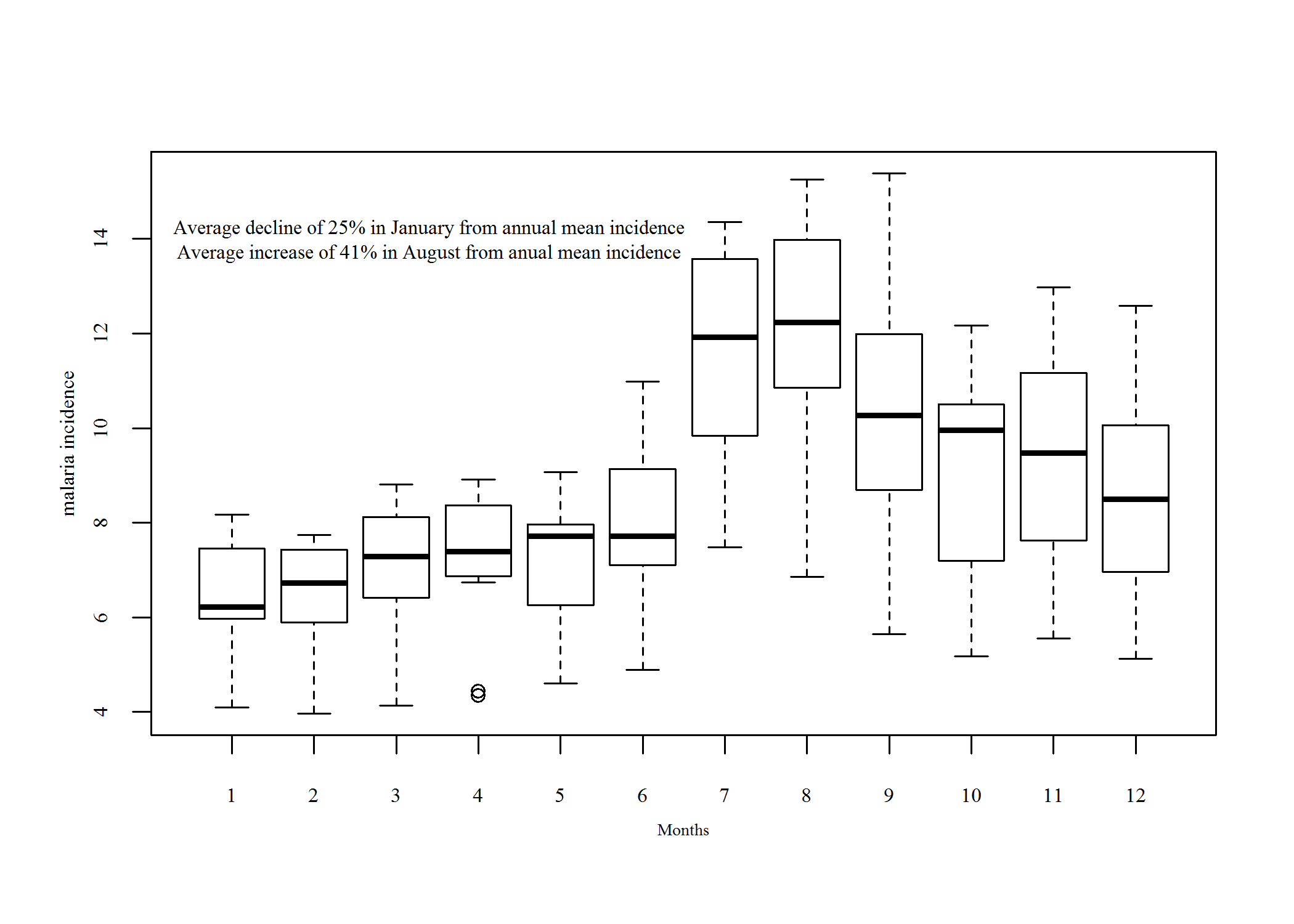

Supplement: S1 Fig — (TIF) [file pone.0149126.s001.tif]
