# Supplementary material for: Trends in Malaria in Odisha, India—An Analysis of the 2003–2013 Time-Series Data from the National Vector Borne Disease Control Program
Source: PLoS One. 2016 Feb 11;11(2):e0149126. doi: 10.1371/journal.pone.0149126 (PMC4750863; doi:10.1371/journal.pone.0149126)
Supplement: S1 File — (PDF) [file pone.0149126.s002.pdf]

CERTIFICATE OF ENGLISH EDITING

This document certifies that the paper listed below has been edited to ensure that the language is clear and free of errors. The edit was performed by professional editors at Editage, a division of Cactus Communications.The intent of the author's message was not altered in any way during the editing process. The quality of the edit has been guaranteed, with the assumption that our suggested changes have been accepted and have not been further altered without the knowledge of our editors.

Title Of The Paper

Trends in Malaria in Odisha, India—An Analysis of the 2003–2013 Time-Series Data from the National Vector Borne Disease Control Program

Authors

Ashirbad Pradhan, Anita Anasuya, Madan M Pradhan, AK Kavitha, Priyanka Kar, Krushna C Sahoo, Pinaki Panigrahi, Ambarish Dutta

Job Code

AMDUT_1

editage by CACTUS

Signature

Nikesh Gosalia,
Vice President, Author Services, Editage

Date of Issue
August 14, 2015

Editage, a brand of Cactus Communications, offers professional English language editing and publication support services to authors engaged in over 500 areas of research. Through its community of experienced editors, which includes doctors, engineers, published scientists, and researchers with peer review experience, Editage has successfully helped authors get published in internationally reputed journals. Authors who work with Editage are guaranteed excellent language quality and timely delivery.

CACTUS®

Contact Editage

Worldwide	Japan	Korea	China	Brazil	Taiwan
request@editage.com	submissions@editage.com	submit-korea@editage.com	fabiao@editage.cn	inquiry.brazil@editage.com	submitjobs@editage.com
+1 877-334-8243	+81 03-6868-3348	1544-9241	400-005-6055	0800-892-20-97	02 2657 0306
www.editage.com	www.editage.jp	www.editage.co.kr	www.editage.cn	www.editage.com.br	www.editage.com.tw
