# Supplementary material for: Trends in Malaria in Odisha, India—An Analysis of the 2003–2013 Time-Series Data from the National Vector Borne Disease Control Program
Source: PLoS One. 2016 Feb 11;11(2):e0149126. doi: 10.1371/journal.pone.0149126 (PMC4750863; doi:10.1371/journal.pone.0149126)
Supplement: S2 File — (DOCX) [file pone.0149126.s003.docx]

**Trends in Malaria in Odisha, India—An Analysis of the 2003–2013 Time-Series Data from the National Vector Borne Disease Control Program**

Ashirbad Pradhan**^1¶^_,_** Anita Anasuya**^2¶^_,_** Madan M Pradhan**^3&^_,_** AK Kavitha**^1&^**_,_ Priyanka Kar**^3&^_,_** Krushna C Sahoo**^1&^_,_** Pinaki Panigrahi**^4&^,** Ambarish Dutta**^1¶*^**

**^1^** Centre for Disease Epidemiology and Surveillance, Asian Institute of Public Health, Bhubaneswar, Odisha, India

**^2^** Department for International Development, United Kingdom supported Technical and Management Support Team, Bhubaneswar, Odisha, India

**^3^** National Vector Borne Disease Control Programme, Department of Health and Family Welfare, Government of Odisha, Bhubaneswar, Odisha, India

**^4^** Center for Global Health and Development, College of Public Health, University of Nebraska Medical Center, Omaha, United States of America

**^*^Corresponding author:**

E-mail: [adutta@aiph.ac.in](mailto:adutta@aiph.ac.in) (AD)

¶ These authors contributed equally to this work.

**&** These authors also contributed equally to this work.

# Abstract

## Background

Although Odisha is the largest contributor to the malaria burden of India, no systematic study has examined its malaria trends. This study aimed to estimate and predict the spatio-temporal trends in malaria in Odisha, against the backdrop of the various anti-malaria strategies implemented in the state.

## Methods

Data on malaria incidence and blood examination (2003–2013) were obtained from the National Vector Borne Disease Control Program (NVBDCP). Blood examination-adjusted time-trends in malaria incidence were estimated for the 2003–2013 time-series data and predicted for 2014–2016. An interrupted time series analysis using segmented regression was conducted to compare the disease trends between the pre (2003–2007) and post-intensification (2009–2013) periods because most of the anti-malaria activities were intensified in 2008. Key-informant interviews of state stakeholders were used to collect the information on the various anti-malaria strategies adopted in the state.

## Results

The annual malaria incidence of Odisha declined from 10.82/1000 to 5.28/1000 during 2003–2013 (adjusted annual decline: -0.54/1000, 95% CI: -0.78 to -0.30). However, the annual blood examination rate remained almost unchanged from 11.25% to 11.77%. The key-informants revealed the intensification of anti-malaria initiatives in 2008, including long-lasting insecticidal nets, new diagnostic techniques and treatment, training of front-line health-workers, and promotional campaigns, which led to a more rapid decline in malaria incidence during 2009–2013 as compared to that in 2003–2007 [adjusted decline: -0.83 (-1.30 to -0.37) and -0.27 (-0.41 to -0.13), respectively]. There was a significant difference in the two temporal slopes, i.e., -0.054 (-0.10 to -0.002, p=0.04) per 1000 population per month, between the pre and post-intensification periods, indicating almost a 200% greater decline in the post-intensification period. The decline was the highest in the districts with maximum malaria burden. However, seven southern high-burden districts continued to be in that zone, making the achievement of malaria elimination (incidence <1/1000) unlikely by 2017.

## Conclusion

The anti-malaria strategies in Odisha, especially their intensification since 2008, have helped the state improve its malaria situation in recent years. These successful measures need to be sustained and perhaps intensified further for eliminating malaria from Odisha.

# Introduction

Being a major contributor to human morbidity, mortality, and economic adversity, malaria is a significant public health problem in India[1,2]. With year-round ubiquitous presence in the sub-continent, it exacts an unacceptable toll on the health of people of all ages[3]. As per the World Malaria Report (WMR) 2013, India contributed to about 52% of the two million confirmed malaria cases in South-East Asia.

India’s varied geography, ecological diversity, and climatic variability make it an ideal place for the widely-spread mosquito vectors to breed and transmit malaria parasites[4], albeit with varied intensity in different parts of the country[5]. The north-eastern, central, and eastern states of India are regarded as high malaria transmission zones accounting for nearly 80% of the total malaria incidence and deaths reported in the country[6,7]. Among these, the worst affected state is Odisha[8].

Given the gravity of the problem, multifarious malaria containment strategies have been implemented periodically in India. The inception of the National Malaria Control Programme and National Malaria Eradication Programme in the 1950s[9], which focused mainly on Indoor Residual Spraying (IRS) with diethyl-dichloro-trichloroethane (DDT) and treating patients with chloroquine[10], yielded unprecedented results by quickly reducing the national malaria burden to 0.1 million cases and no deaths in 1965, from an estimated 75 million cases and 0.8 million deaths in 1947, putting India on the verge of malaria eradication[11,12]. However, this dramatic success proved to be counter-productive as many malaria-specific public health resources were withdrawn and infrastructure was prematurely dismantled in the country[12,13]. In the 1970s, malaria resurged and remained more or less static with about 2–3 million episodes up to the latter part of the 1990s[10].

The combat against malaria intensified in the 1990s with the introduction of new strategies, often through various externally-aided projects like the Enhanced Malaria Control Project in 1997 and Intensified Malaria Control Programme in 2005, which were supported by the World Bank and The Global Fund to Fight AIDS, TB and Malaria (GFATM), respectively. The main components of the intensified anti-malaria efforts included integrated vector control measures, use of newer techniques for early diagnosis and effective treatment, service decentralization, behavior change communication (BCC), improved surveillance, and inter-sectorial convergence. Since 2002, these strategies are being implemented under the National Vector Borne Diseases Control Programme (NVBDCP)[14,15]. Furthermore, in view of the availability of more effective antimalarial drugs and proven malaria control strategies, two consecutive revisions in the national drug policy in 2007 and 2008, marked by unprecedented utilization of resources and control efforts to a level not seen before, revitalized the country’s malaria control efforts[9].

Consequently, India witnessed a considerable decline in the malaria burden from nearly 3.04 million reported cases and 1010 deaths in 1996 to 0.85 million cases and 519 deaths in 2013[15,16]. This progress can be attributed to increased political commitment, concerted national and international efforts, and tremendous expansion in financing through various mechanisms[15,17].

Odisha, with only about 4% land area and 3% population of India, accounted for 26.9% of India’s malaria cases, and about 17.6% of all reported deaths in 2013[16]. Over the years, Odisha has also continued its combat against malaria in convergence with the previously mentioned national anti-malaria strategies, often through bilaterally-supported projects like the Odisha Health Sector Plan (OHSP), 2007–08 that was funded by the Department for International Development (DFID), United Kingdom (UK)[18].

As stated above, the malaria situation in India has improved considerably over the years, notwithstanding the various pitfalls in the anti-malaria activities mounted by the public health system of the country. However, the malaria trends in Odisha have hardly been studied. Hence, this study aimed to examine the spatio-temporal situation of malaria in the state during 2003–2013, in the light of the various anti-malaria measures undertaken. It also aimed to predict the future trends related to the disease, so as to inform anti-malaria policy. Additionally, this study evaluated the disease trend in the state, before and after the introduction of the revised anti-malaria drug policy (2007), to detect any significant effect greater than the secular trend.

# Materials and Methods

## Time-series data

District-wise (30 districts) monthly malaria surveillance data (2003–2013) obtained from the NVBDCP, Odisha, were converted into the annualized rates of malaria incidence and blood examination, and structured as multiple month-wise time-series. The mid-year district-wise population was used for computing these rates derived from the national censuses (2001 and 2011) and for accounting for the growth in the population. Blood examination included both microscopy-based blood slide examination and blood samples examined by Rapid Diagnostic Tests (RDTs). Data entry and analysis were conducted using the Epi Info v.7.1.4 and R v3.0.1 software, respectively[19].

## Qualitative information

Sixteen semi-structured consented key-informant interviews on anti-malaria strategies adopted by the state during 2003–2013 were conducted with stakeholders from the NVBDCP, Odisha and OHSP in order to explore the trends in detail.

## Ethics statement

The data used in our analysis were district-aggregate programmatic data of the NVBDCP, collected from the Odisha bureau of the program. These were routinely collected by the program information system from 2003 through 2013, before the analysis was undertaken in 2014. No individual data was analyzed in our study; hence, there was no scope or need for anonymization or de-identification of data. Thus, the ethical consideration was deemed inapplicable in this context.

## Data analysis

### Estimation of trend and seasonality

The time-series data were decomposed to describe their seasonality and secular trends. The trends were graphically examined using raw time-series data, centered moving averages, locally weighted, and linear regression lines. The decomposed seasonality was further explored to determine monthly fluctuations from the mean.

The unadjusted annual trends were initially estimated using generalized least square regression[20,21] models, including time as the only explanatory variable. A correlogram and partial correlogram of the residuals were graphically plotted with each time point as the lag. The underlying structures of the serial dependence of the residuals were examined and their order of Auto Regression and Moving Averages parameters were estimated. These parameters were then included in the successive models to control for the serial dependence of the residuals. A multi-variable model adjusted for time-trends in malaria incidence was then estimated after controlling for blood examination rates.

### Analysis of the pre and post-intensification periods

Important strategic changes in the national and state anti-malaria programs, and roll-out and scaling-up of those new anti-malaria interventions, along with the intensification of some of the ongoing strategies occurred in the state during 2008–2009. Hence, we analyzed the malaria time-series data after dividing them into two periods: first phase ranging from 2003 to 2007, denoting the pre-intensification period, and second phase ranging from 2009 to 2013, denoting the post-intensification period. The year 2008 was excluded from this analysis of pre and post-intensification periods as this was the year considered for ramping up the activities. The time-trends between these two periods were compared using a segmented regression of the interrupted time-series[22–24]. We also conducted a sensitivity analysis using the same methodology, after including the year 2008 in the pre-intensification period[22]. Thus, the time-point at which maximum significant change(s) occurred after the intensification of anti-malaria activities was identified by means of change-point analysis[25].

### Analysis of the districts stratified by the baseline disease burden

Districts were stratified as per their annual malaria incidence in 2003 (S1 Table). The districts with an annual blood examination rate of <10% were corrected as per the NVBDCP guidelines and then stratified into the following four clusters: 0–1.9=“Low,” 2–4.9=“Moderate,” 5–9.9=“High” and >10=“Very High” (VH), the numbers denoting the annual malaria incidence in 2003. Then the trends in malaria incidence in these four clusters were estimated using the same process as described above.

### Prediction

The malaria incidence was predicted for the next three years (2014–2016) using historical disease incidence rates (2003–2013) and the Holt-Winters exponential smoothing forecasting models. The appropriateness of the predictive model was checked using the Box-Ljung test.

### Spatial distribution of malaria

The districts stratified as per the aforesaid cut-off points were color-coded in the state-map once at baseline (2003) and then for 2008 and 2013, to display the progress of each individual district with regards to their disease burden.

### Analysis of the qualitative data

The information from the key-informants was noted during the interview by one researcher while the other conducted the interview. After each interview, the researcher debriefed the data and adopted a new guide based on the previous findings. The latent content analysis methods[26] were used. Meaning units were then selected from the notes and coded. Similar codes were clustered together and merged into categories. The main theme and emergent concepts were developed based on the similarities and interpretation of codes and categories.

# Results

During the eleven years (2003–2013), the annual malaria incidence of Odisha decreased from 10.82 per 1000 population in 2003 to 5.28 in 2013, whereas the annual blood examination rate remained almost unchanged from 11.25% in 2003 to 11.77% in 2013 (Table 1).

**Table 1. Annual Malaria Incidence and Blood Examination Rate, 2003–2013, Odisha.**

| **Year** | **Malaria cases detected by the NVBDCP** | **Blood slides examined by the NVBDCP** | **Population** | **Annual Malaria Incidence per 1000 population** | **Annual Blood Examination Rate (%)** |
| --- | --- | --- | --- | --- | --- |
| **2003** | 409445 | 4256451 | 37833200 | 10.82 | 11.25 |
| **2004** | 398305 | 4188029 | 38347469 | 10.39 | 10.92 |
| **2005** | 391830 | 4770794 | 38861739 | 10.08 | 12.28 |
| **2006** | 376214 | 4920147 | 39376009 | 9.55 | 12.50 |
| **2007** | 364318 | 4805306 | 39890279 | 9.13 | 12.05 |
| **2008** | 343778 | 4790798 | 40404549 | 8.51 | 11.86 |
| **2009** | 359493 | 4826635 | 40918818 | 8.79 | 11.80 |
| **2010** | 364432 | 4971009 | 41433088 | 8.80 | 12.00 |
| **2011** | 308374 | 4659729 | 41947358 | 7.35 | 11.11 |
| **2012** | 248948 | 4555739 | 42633052 | 5.84 | 10.69 |
| **2013** | 227990 | 5078508 | 43147321 | 5.28 | 11.77 |

## Trends in malaria in Odisha, 2003–2013

There was a significant annual decline in the malaria incidence in the state, the linear trend being -0.49 per 1000 population (95% CI: -0.60 to -0.37), which increased to

-0.54 (-0.78 to -0.30) after adjustment for blood examination. In contrast, the blood examination rate underwent hardly any change with a change of -0.02 percentage points (-0.12 to 0.08) over the same period (Fig. 1).

**Fig 1. Trends in malaria incidence and blood examination from 2003–2013, Odisha.**

### Seasonal variability

The highest peak of this seasonal disease in almost all the years was observed during July–August, showing a 41% increase in the incidence as compared to the annual mean. The minimum malaria incidence was observed during January, which was 25% less than the annual mean incidence (S1 Fig.). However, since 2008, the peak incidence of the disease showed a slight shift towards August.

## Trends during the pre-intensification vs. post-intensification periods (2003–2007 vs. 2009–2013)

The overall linear slope of decline in the malaria incidence between 2003 and 2007 was considerably flatter than that for 2009–2013 (Fig. 2).

**Fig 2. Malaria incidence during 2003–2007 and 2009–2013, Odisha.**

The estimated annual decline for 2003–2007 was -0.19 per 1000 population (-0.85 to 0.46, p=0.56), which increased to -0.27 per 1000 population (-0.41 to -0.13) after adjustment for blood slide examination. In comparison, the annual decline during the period of 2009–2013 was -0.81 (-1.46 to -0.18), which slightly increased to -0.83 (-1.30 to -0.37) after adjustment (Table 2). Further, the blood examination rate in the state increased by 0.34 percentage points (0.12 to 0.56, p=0.013) annually during 2003–2008, whereas the change during 2009–2013 was not significant.

**Table 2. Trends in Malaria Incidence, Odisha.**

|  | **Unadjusted trend estimate* of malaria incidence** | **Trend estimate* of malaria incidence adjusted for blood examination rate** |
| --- | --- | --- |
| **Odisha, 2003–2013** | -0.49 (-0.60 to -0.37), p<0.0001 | -0.54 (-0.78 to -0.30), p<0.0001 |
| **Two periods** | | |
| Odisha, 2003–2007 | -0.19 (-0.85 to 0.46), p=0.56 | -0.27 (-0.41 to -0.13), p<0.0001 |
| Odisha, 2009–2013 | -0.81 (-1.46 to -0.18), p=0.015 | -0.83 (-1.30 to -0.37), p<0.0001 |
| **Strata as per disease burden at baseline, 2003** | | |
| Very High | -0.78 (-1.06 to -0.49) , p<0.0001 | -0.69 ( -0.94 to -0.33) , p<0.0001 |
| High | -0.34 (-0.44 to -0.25) , p<0.0001 | -0.44 (-0.48 to -0.40) , p<0.0001 |
| Moderate | -0.24 (-0.29 to -0.19) , p<0.0001 | -0.27 (-0.31 to -0.23) , p<0.0001 |
| Low | -0.04 (-0.08 to -0.01), p=0.013 | -0.04 (-0.08 to 0.00), p= 0.06 |

*Trend estimates are per 1000 population per year

The segmented regression of the interrupted time-series demonstrated a significant difference in the temporal slopes between the pre-intensification (2003–2007) and post-intensification (2009–2013) periods, separated by the intensification phase of 2008. The difference in the two slopes was -0.054 (-0.10 to -0.002. p=0.04) per 1000 population per month, indicating almost a 200% greater decline in the post-intensification period as compared to that in the pre-intensification period (Fig. 2). The sensitivity analysis with the inclusion of 2008 in the pre-post analysis only changed the results marginally.

The intensification phase of 2008 was followed by a surge in case detection between 2009 and 2010 (Fig. 1). This was followed by a steep decline from 2011, the sharp decline somewhat slowing down in 2012, as evident from the linear smoothers in Fig. 2. The change-point analysis showed that the maximum change during 2003–2013 occurred in the month number 102 in the time-series, which was June 2011. During that month, the maximum decline was experienced, as also evident from the visual exploration of the time-series (Fig. 2)

## Trends in the districts stratified by the baseline disease burden

The annual relative decline in the malaria incidence in Low burden cluster of districts had a lesser gradient than did the other three clusters of districts, where the declines were comparable (Fig. 3). The blood examination rate-adjusted gradients were -0.69 (-0.94 to -0.33), -0.44 (-0.48 to -0.40), -0.27 (-0.31 to -0.23), and -0.04 (-0.08 to 0.00) for the VH, High, Moderate and Low clusters, respectively. For the first three clusters, the annual decline, although different in absolute terms, was actually very similar in terms of the decline relative to their baseline.

**Fig 3. Trends in malaria in the districts stratified by their baseline malaria burden (2003), Odisha.**

The blood examination rate registered a decline in the VH cluster over the years, with the annual percentage point decline estimated to be -0.29 (-0.45 to -0.14). The other three clusters experienced increase in the blood examination rates during these eleven years, the annual estimates being 0.20 (0.39 to 9.08), 0.18 (0.05 to 0.30), and 0.28 (0.24 to 0.34) for the High, Moderate and Low clusters, respectively.

## Prediction

The forecast for the next three years (2014–2016) (Fig. 4) showed that as compared to the rapid decline of 2011–2012, the decline was likely to slow down. This implies that the elimination level of annual malaria incidence of <1/1000 might not be achieved by 2017 for the whole state, which is the current goal of the NVBDCP. The districts in the Low and Moderate incidence clusters had already reached this elimination level, and the High incidence cluster is quite likely to reach it by 2017 (Fig. 2). The VH incidence cluster started out at very high levels, and showed the steepest decline over the eleven year period. However, as per the current state, it is unlikely to reach the elimination level by 2017.

**Fig 4. Forecasting malaria incidence for 2014–2016 based on the trends from 2003–2013, Odisha.**

## Spatial distribution of malaria

Malaria continued to ravage the seven southern districts of Kandhamal, Kalahandi, Rayagada, Koraput, Nawarangpur, Nuapada, and Malkangiri, and two central districts of Sambalpur and Deogarh, which were consistently in the VH incidence zone from 2003 through 2013. More palpable success had been achieved in north-western districts, some of which have moved from the VH to other less-burden clusters. The five coastal districts remained in the Low cluster throughout the study period (Fig. 5).

**Fig 5. Annual malaria incidence (2003, 2008, and 2013), Odisha.**

## Increased administrative and political commitment to reduce the malaria burden in the state

The information from key-informants revolved around various anti-malaria measures undertaken during 2008–2013 vis-à-vis that of 2003–2007. As per the perception of the informants, during 2003–2007, no new strategy other than the routinely implemented ones were used against the malaria challenge in the state, leading to the deceleration of the consolidation of the success achieved in the early nineties. Interviewees particularly revealed a massive roll-out of artemisinin-based combination therapy (ACT) and RDT replacing and supplementing microscopy-based blood examination by the NVBDCP in the state, especially in the VH districts during 2008. These steps were implemented in compliance with the revised National Drug Policy, 2007 and surge in other existing and new activities to accomplish this scale-up, and were sustained thereafter. The main theme that emerged from the key informant interviews was “Increased political and administrative commitment to reduce the malaria burden in the state” since 2008, which evolved from the categories explained in Table 3. The interviewees also perceived that the surge in the programmatic inputs had been instrumental in improving the malaria situation in the state.

**Table 3 Anti-malaria Inputs Rolled out from 2008–2013, Odisha.**

| **Theme** Increased administrative and political commitment to reduce the malaria burden in the state | | | | | |
| --- | --- | --- | --- | --- | --- |
| **Categories** | Vector control | Case detection and management | Behavioral Change Communication (BCC) strategies | Human resources | Supportive measures |
| **Codes** | Free distribution of Long Lasting Insecticidal Nets (LLINs) in high endemic districts  Successful implementation of the “Mo Masari scheme”^a^  Wider Indoor Residual Spray (IRS) coverage | Set up of fever treatment depots (FTD) at the community level  Sufficient supply and wider coverage of Rapid Diagnostic Kits (RDKs) and artemisinin-based combination therapy (ACTs) | Innovative BCC campaigns promoting the use and maintenance of bed-nets, e.g., “Nidhi Mousa To Masari Ne”^b^  Social mobilization drives through the “Nidhi Ratha”^c^ and folk theatre resulting in improvement in treatment seeking behavior  Health messages transmission through interpersonal communication by frontline health workers | A multi-disciplinary strong technical team at the National Vector Borne Disease Control Program (NVBDCP)  Deployment of Accredited Social Health Activists (ASHAs) in anti-malaria activities  Capacity building of ASHAs through training on malaria diagnosis using Rapid Diagnostic Tests (RDTs), and anti-malaria drug administration  Provision of malaria technical supervisors, vector-borne disease consultants, and trained health workers for malaria | Highly supportive bureaucratic and administrative environment  Regular fixed day technical committee meetings  Multiple stakeholders’ involvement  Financial and technical support from the DFID, World Bank, GFATM, and World Health Organization  Government willingness on extensive investment on LLINs  Strengthening of malaria surveillance and information systems by using standardized formats |

^a^ “Mo Masari” or “my mosquito net” is an endeavor by the Government of Odisha to protect all pregnant mothers, and under five and tribal school children in highly endemic areas

^b^ A pre-publicity BCC campaign to generate demand for mosquito nets and demonstration of their use during LLINs distribution

^c^ “Nidhi Ratha” the name of a chariot used as a part of the “Nidhi Mousa To Masari Ne” campaign for imparting messages on malaria prevention and control; and usage of LLIN by organizing folk theatres in Odia language and distributing leaflets throughout its journey

# Discussion

The present study examined the temporal trend in malaria morbidity in Odisha from 2003–2013, with the state achieving a 44.32% reduction in confirmed malaria infections (an average decline of approximately 5% every year from its annual incidence in 2003, when it was almost eleven per 1000 population). The maintenance of a steady blood examination rate in the state during this period, a key indicator of effective malaria surveillance[2], was indeed a driver for this decline. However, the average annual decline in the incidence during 2009–2013 was steeper than that during 2003–2007, notwithstanding the peak in malaria case detection in 2009–2010. This difference in temporal slopes of malaria incidence between the pre-intensification and post-intensification periods were formally tested statistically using the robust method of segmented regression of interrupted time series, which has been successfully used to examine the impact of policy and intervention in a quasi-experimental design setting[21].

The peak in 2009–2010 signifies the increase in case detection with the large-scale surge in anti-malaria activities preceding this period, i.e., in 2008. Indeed, the effective management of a large number of detected cases within a short time-period using ACT led to a rapid contraction of the reservoir of malaria parasites in the community. This, in co-ordination with the other intensified efforts undertaken during this period, ushered in the sharp decline in the malaria incidence from 2011. This was also formally endorsed by the change-point analysis of our data, which indicated maximum decline, and revealed the break-point to be during June 2013. This sharp decline accounted for the steeper overall decline observed during 2009–2013, i.e., in the post-intensification period. The introduction and intensification of various effective anti-malaria approaches during this period included integrated vector control involving the distribution and promotion of long-lasting insecticidal nets (LLINs), early case detection and management with the large-scale roll-out of RDTs and ACTs, standardized monitoring and evaluation strategies, introduction of innovative behavioral change communication approaches, and provision of additional manpower along with training and deployment of existing staff and volunteers for anti-malaria activities. These were possible due to the increased political and administrative commitment from the state and national policy-makers, with adequate support from international agencies. Our study underscored that these measures led to favorable outputs, which as was evident from the peak-decline pattern during 2009–2010 further lowering the overall malaria burden in the state considerably during 2009–2013. This was in contrast to the slower decline experienced during the pre-intensification period of 2003–2007, despite a slight rise in blood slide examination rates. If not a surge, this at least indicated the maintenance of the same level of emergence of fever cases in the community due to the absence of a rapid decline in the malaria case burden. The intensification of the existing measures and implementation of new measures such as utilization of RDT and ACT, Accredited social health activists’ (ASHAs) training, and free distribution of LLINs, gained momentum after that period, with the occurrence of several major technical changes in the malaria drug policy (2007), as revealed by program managers from various levels of the NVBDCP in the state.

As a post-hoc analysis to validate the information on the intensification of the anti-malaria activities during 2008–2009, we explored the Lot Quality Assurance Sampling (LQAS) survey results published recently by Valadez et al. The LQAS was used to evaluate the coverage and performance of various programmatic inputs by the NVBDCP for the mid-term course-correction[15]. It showed a remarkable increase in various programmatic components in the initial districts, such as protection of adults and children (27% and 42% increase, respectively) through the use of LLINs, improved treatment-seeking behavior, and increased treatment of diagnosed cases (average increase of 63%). However Valadez et al. also underscored the weaknesses in the program, such as lack of knowledge regarding maintenance of LLIN, thus compromising their lifespan, and sporadic stock-outs of RDTs in some districts*.*

The sharp decline in the incidence from 2011 slowed down in 2012 and 2013, as was also reflected in the prediction for the next three years (2014–2016), thus raising concerns for possible deceleration of the success achieved in the state. This “slowing down” was also perhaps due to the non-renewal of the LLINs in the state after the expiry of the shelf-life of the current crop, majority of which were distributed in 2008[27]. However, the comparative contribution of each individual specific anti-malaria measure to this decline, using district-wise analyses, could not be explored quantitatively because of the absence of robust data owing to reporting inconsistency and incompleteness. Thus, we relied on the qualitative information to suggest that the considerable decline in the malaria incidence in the state was due to the intensification of malaria control interventions. Despite this, the goal of achieving a state-wide malaria incidence of <1/1000 by 2017[15] is unlikely to be met as the seven southern districts continued to be in the VH (>10/1000) zone consistently over the last 11 years, though each of these districts achieved remarkable decline as compared to their higher baseline burdens. The districts belonging to the other clusters either had achieved or are likely to achieve this elimination target by 2017. Sustaining or even heightening the current anti-malaria measures with proven past success as demonstrated in our analysis, with targeted resource-allocation for VH burden districts or blocks, may be the necessary strategy for the “last push” to bring malaria to the elimination level in the whole state. This would offset any trend of slackening in surveillance in those areas, as might be evident by the decline of the blood examination rate in the VH cluster. However, perhaps much of that decline in blood examination could be ascribed to the substantial reduction of fever cases in the community due to the rapid decline in the malaria burden and also the change in the NVBDCP policy on blood examination, whereby many obvious non-malarial causes of fever were excluded[28]. Additionally, this decline in the blood examination rate in this cluster is perhaps a testament to the rapid decrease in malarial fever cases in these communities.

The limitation of the present study could be the use of secondary surveillance data of the NVBDCP, which included the cases detected and reported by the program. This may not have been a true reflection of all the malaria cases in the community, as shown by other studies in India[29,30]. Those “left-out” by the program might be seeking care from other sources such as private or non-formal medical systems. Despite this, it could be argued that such an underestimation due to the non-notification from the private sector is unlikely to influence our time-trend estimates substantively because the portion of the “uncovered” population was unlikely to have increased with time. Hence, the decline we notice is very unlikely to be due to decreasing program coverage but was achieved by the mounting of effective preventive and curative strategies by the NVBDCP. Further, the pool of “uncovered” malaria cases would have reduced over time as many measures were successfully undertaken to increase the access to the NVBDCP in the state. Moreover, the strength of this study is that the decline of malaria incidence as observed was adjusted for a key operational variable, i.e., blood examination rate, which accounted for fluctuations in program surveillance. The blood examination rate remaining largely unchanged during the period of our analysis, this indicates that program surveillance did not undergo any major decline during the study period. Further, the intensification of anti-malaria activities undertaken in 2008 saw a major overhaul of microscopy-based blood examination strategy, as it was largely replaced by RDT. This proved to be more effective in the diagnosis of malaria cases, evident from the resulting surge of cases during 2009–2010.

The other limitation of this study was the lack of quantitative data regarding the intensification of the anti-malaria activities in 2008. However, the exploration of the situation using qualitative techniques has pointed towards the intensification of many anti-malaria activities in 2008. Additionally, the intensification of many of such new and ongoing efforts such as political commitment and supervisory efforts are often better explored qualitatively.

# Conclusion

To conclude, our study, perhaps for the first time to our knowledge, has systematically estimated the malaria trends in Odisha and has shed light on the substantial decline in the malaria incidence in the state during the last 11 years (2003–2013). The bulk of this decline was achieved between 2009–2013, especially in the districts with the maximum disease burden, mainly owing to the initiation, scaling-up, and intensification of existing and various new anti-malaria strategies since 2008 in the state. Nevertheless, many districts continue to have a very high burden of malaria, and hence, a stagnation of the success or resurgence of the problem in those areas and the neighboring regions cannot be ruled. Therefore, the activities and control measures that have helped achieve the success in past need to be sustained or even increased in future.

# Acknowledgements

We thank the officials from the National Vector Borne and Disease Control Programme, Odisha and Odisha Health Sector Plan for their co-operation in the qualitative interviews. We would also like to thank the staff from the National Vector Borne and Disease Control Programme, Odisha for their assistance in providing malaria surveillance data for the study.

# References

1. Dhingra N, Jha P, Sharma VP, Cohen AA, Jotkar RM, Rodriguez PS, et al. Adult and child malaria mortality in India: A nationally representative mortality survey. Lancet [Internet]. 2010 Nov 20 [cited 2014 Jun 6];376(9754):1768-74. Available from: http://www.pubmedcentral.nih.gov/articlerender.fcgi?artid=3021416&tool=pmcentrez&rendertype=abstract

2. Kumar A, Valecha N, Jain T, Dash AP. Burden of malaria in India: Retrospective and prospective view. Am J Trop Med Hyg [Internet]. 2007 Dec 1 [cited 2014 Mar 8];77(6_Suppl):69-78. Available from: http://www.ajtmh.org/content/77/6_Suppl/69.long

3. Strategic Action Plan for Malaria Control in India [Internet]. India: Directorate of National Vector Borne Disease Control Programme; 2007:1-84. Available from: http://www.nvbdcp.gov.in/malaria-new.html

4. Das A, Anvikar AR, Cator LJ, Dhiman RC, Eapen A, Mishra N, et al. Malaria in India: the Center for the Study of Complex Malaria in India. Acta Trop [Internet]. 2012 Mar [cited 2014 May 4];121(3):267-73. Available from: http://www.pubmedcentral.nih.gov/articlerender.fcgi?artid=3294179&tool=pmcentrez&rendertype=abstract

5. Singh N, Singh MP, Wylie BJ, Hussain M, Kojo YA, Shekhar C, et al. Malaria prevalence among pregnant women in two districts with differing endemicity in Chhattisgarh, India. Malar J [Internet]. 2012 Jan [cited 2014 May 15];11(1):274. Available from: http://www.pubmedcentral.nih.gov/articlerender.fcgi?artid=3489539&tool=pmcentrez&rendertype=abstract

6. Patil RR, Kumar RK. World bank EMCP malaria project in Orissa, India - A field reality. Trop Parasitol [Internet]. 2011 Jan [cited 2014 May 15];1(1):26-9. Available from: http://www.pubmedcentral.nih.gov/articlerender.fcgi?artid=3593465&tool=pmcentrez&rendertype=abstract

7. Annual Report to the People on Health [Internet]. Ministry of Health and Family Welfare, Government of India; 2011:67. Available from: http://mohfw.nic.in/WriteReadData/l892s/6960144509Annual Report to the People on Health.pdf

8. Sahu S, Gunasekaran K, Vanamail P, Jambulingam P. Seasonal prevalence and resting behavior of Anopheles minimus Theobald & An. fluviatilis James (Diptera: Culicidae) in east-central India. Indian J Med Res [Internet]. 2011 [cited 2014 May 22];133(6):655-61. Available from: http://icmr.nic.in/ijmr/2011/june/0612.pdf

9. Anvikar AR, Arora U, Sonal GS, Mishra N, Shahi B, Savargaonkar D, et al. Antimalarial drug policy in India: past, present & future. Indian J Med Res [Internet]. 2014 Feb;139(February):205-15. Available from: http://www.pubmedcentral.nih.gov/articlerender.fcgi?artid=4001331&tool=pmcentrez&rendertype=abstract

10. Dash A, Valecha N, Anvikar AR, Kumar A. Malaria in India: challenges and opportunities. J Biosci [Internet]. 2008 Nov;33(4):583-92. Available from: http://www.ncbi.nlm.nih.gov/pubmed/19208983

11. Shiv L, G.S S, P.K P. Status of Malaria in India. J Indian Acad Clin Med [Internet]. 5(1):19-23. Available from: http://medind.nic.in/jac/t00/i1/jact00i1p19.pdf

12. Sharma VP. Battling malaria iceberg incorporating strategic reforms in achieving Millennium Development Goals and malaria elimination in India. Indian J Med Res [Internet]. 2012 [cited 2014 Jun 3];136(December):907-25. Available from: http://icmr.nic.in/ijmr/2012/december/Centenary Review Article.pdf

13. Sharma VP, Mehrotra KN. Malaria resurgence in India: a critical study. Soc Sci Med [Internet]. 1986 Jan [cited 2014 Jul 15];22(8):835-45. Available from: http://www.ncbi.nlm.nih.gov/pubmed/3749959

14. Operational Manual for Implementation of Malaria Programme 2009 [Internet]. India: Directorate of National Vector Borne Disease Control Programme; 2009:1-275. Available from: http://nvbdcp.gov.in/Doc/malaria-operational-manual-2009.pdf

15. Strategic Action Plan for Malaria Control in India [Internet]. India: Directorate of National Vector Borne Disease Control Programme; 2012:1-114. Available from: http://nvbdcp.gov.in/Round-9/Annexure-2  Strategic action plan.pdf

16. National Vector Borne Disease Control Programme (NVBDCP) [Internet]. [cited 2014 May 14]. Available from: http://nvbdcp.gov.in/malaria3.html

17. Bhatia R, Rastogi RM, Ortega L. Malaria successes and challenges in Asia. J Vector Borne Dis [Internet]. 2013 [cited 2014 May 15];50(December):239-47. Available from: http://www.searo.who.int/entity/world_health_day/2014/01-1434_rev.pdf

18. Odisha Health Sector Plan Policy and Activity Briefs [Internet]. 2012 [cited 2014 May 20]:1-42. Available from: http://www.nrhmorissa.gov.in/writereaddata/Upload/Documents/OHSP.pdf

19. R Development Core Team. R: A Language and Environment for Statistical Computing. Vienna, Austria : the R Foundation for Statistical Computing. [Internet]; 2011 [cited 2014 Jun 12]. Available from: http://www.r-project.org/.

20. Fox J. Time-Series Regression and Generalized Least Squares. Appendix to An R and S-PLUS Companion to Applied Regression [Internet]; 2002: 1-8. Available from: http://cran.r-project.org/doc/contrib/Fox-Companion/appendix-timeseries-regression.pdf

21. Dayer MJ, Jones S, Prendergast B, Baddour LM, Lockhart PB, Thornhill MH. Incidence of infective endocarditis in England, 2000–13: a secular trend, interrupted time-series analysis. Lancet [Internet]. 2014 Nov 18 [cited 2014 Nov 19]; Available from: http://www.thelancet.com/article/S0140673614620079/fulltext

22. Taljaard M, McKenzie JE, Ramsay CR, Grimshaw JM. The use of segmented regression in analyzing interrupted time series studies: an example in pre-hospital ambulance care. Implement Sci [Internet]. 2014 Jan [cited 2014 Dec 5];9(1):77. Available from: http://www.pubmedcentral.nih.gov/articlerender.fcgi?artid=4068621&tool=pmcentrez&rendertype=abstract

23. Wagner AK, Soumerai SB, Zhang F, Ross-degnan D. Segmented regression analysis of interrupted time series studies in medication use research. J Clin Pharm Ther [Internet]. 2002;27:299-309. Available from: http://isites.harvard.edu/fs/docs/icb.topic79832.files/L06_Program_Evaluation_2/Segmented_Regression.Wagner.2002.pdf

24. Aregawi M, Lynch M, Bekele W, Kebede H, Jima D, Taffese HS, et al. Time series analysis of trends in malaria cases and deaths at hospitals and the effect of antimalarial interventions, 2001-2011, Ethiopia. PLoS One [Internet]. 2014 Jan [cited 2014 Nov 19];9(11):e106359. Available from: http://www.ncbi.nlm.nih.gov/pubmed/25406083

25. Hinkley DV. Inference about the change-point in a sequence of random variables. Biometrika [Internet]. 1970 Apr 1 [cited 2014 Dec 19];57(1):1-17. Available from: http://biomet.oxfordjournals.org/content/57/1/1.abstract

26. Graneheim UH, Lundman B. Qualitative content analysis in nursing research: concepts, procedures and measures to achieve trustworthiness. Nurse Educ Today [Internet]. 2004 Feb 2 [cited 2014 Jul 9];24(2):105-12. Available from: http://www.nurseeducationtoday.com/article/S0260691703001515/fulltext

27. Valadez JJ, Devkota B, Pradhan MM, Meherda P, Sonal GS, Dhariwal A, et al. Improving malaria treatment and prevention in India by aiding district managers to manage their programs with local information: A trial assessing the impact of Lot Quality Assurance Sampling on program outcomes. Trop Med Int Health [Internet]. 2014 Jul 21 [cited 2014 Aug 12]; Available from: http://www.ncbi.nlm.nih.gov/pubmed/25039710

28. Anvikar A, Arora U, Das B, Dash A., Dhillon GP., Dua V., et al. Guidelines for diagnosis and treatment of malaria in India [Internet]. India: NVBDCP, Government of India; 2009:118. Available from: http://nvbdcp.gov.in/Doc/Guidelines_for_Diagnosis___Treatment.pdf

29. Shah NK, Kumar A, Valecha N. New global estimates of malaria deaths. Lancet [Internet]. 2012 Aug 11 [cited 2014 Mar 29];380(9841):560. Available from: http://www.thelancet.com/journals/a/article/PIIS0140-6736(12)61322-1/fulltext

30. Shah NK, Dhariwal AC, Sonal GS, Gunasekar A, Dye C, Cibulskis R. Malaria-attributed death rates in India. Lancet [Internet]. 2010 Mar 19 [cited 2014 May 15];377(9770):991. Available from: http://www.thelancet.com/journals/a/article/PIIS0140-6736(11)60378-4/fulltext

# References

1. Dhingra N, Jha P, Sharma VP, Cohen A a, Jotkar RM, Rodriguez PS, et al. Adult and child malaria mortality in India: a nationally representative mortality survey. Lancet [Internet]. Elsevier Ltd; 2010 Nov 20 [cited 2014 Jun 6];376(9754):1768–74. Available from: http://www.pubmedcentral.nih.gov/articlerender.fcgi?artid=3021416&tool=pmcentrez&rendertype=abstract

2. Kumar A, Valecha N, Jain T, Dash AP. Burden of Malaria in India: Retrospective and Prospective View. Am J Trop Med Hyg [Internet]. 2007 Dec 1 [cited 2014 Mar 8];77(6_Suppl):69–78. Available from: http://www.ajtmh.org/content/77/6_Suppl/69.long

3. Strategic Action Plan for Malaria Control in India [Internet]. India: Directorate of National Vector Borne Disease Control Programme; 2007 p. 1–84. Available from: http://www.nvbdcp.gov.in/malaria-new.html

4. Das A, Anvikar AR, Cator LJ, Dhiman RC, Eapen A, Mishra N, et al. Malaria in India: the Center for the Study of Complex Malaria in India. Acta Trop [Internet]. Elsevier B.V.; 2012 Mar [cited 2014 May 4];121(3):267–73. Available from: http://www.pubmedcentral.nih.gov/articlerender.fcgi?artid=3294179&tool=pmcentrez&rendertype=abstract

5. Singh N, Singh MP, Wylie BJ, Hussain M, Kojo Y a, Shekhar C, et al. Malaria prevalence among pregnant women in two districts with differing endemicity in Chhattisgarh, India. Malar J [Internet]. Malaria Journal; 2012 Jan [cited 2014 May 15];11(1):274. Available from: http://www.pubmedcentral.nih.gov/articlerender.fcgi?artid=3489539&tool=pmcentrez&rendertype=abstract

6. Patil RR, Kumar RK. World bank EMCP malaria project in Orissa, India - A field reality. Trop Parasitol [Internet]. 2011 Jan [cited 2014 May 15];1(1):26–9. Available from: http://www.pubmedcentral.nih.gov/articlerender.fcgi?artid=3593465&tool=pmcentrez&rendertype=abstract

7. ANNUAL REPORT to the People on Health [Internet]. Ministry of Health and Family Welfare, Government of India. 2011 p. 67. Available from: http://mohfw.nic.in/WriteReadData/l892s/6960144509Annual Report to the People on Health.pdf

8. Sahu S., Gunasekaran K, Vanamail P, Jambulingam P. Seasonal prevalence & resting behavior of Anopheles minimus Theobald & An. fluviatilis James (Diptera: Culicidae) in east-central India. Indian J Med Res [Internet]. 2011 [cited 2014 May 22];133(6):655–61. Available from: http://icmr.nic.in/ijmr/2011/june/0612.pdf

9. Anvikar AR, Arora U, Sonal GS, Mishra N, Shahi B, Savargaonkar D, et al. Antimalarial drug policy in India: past, present & future. Indian J Med Res [Internet]. 2014 Feb;139(February):205–15. Available from: http://www.pubmedcentral.nih.gov/articlerender.fcgi?artid=4001331&tool=pmcentrez&rendertype=abstract

10. Dash A, Valecha N, Anvikar AR, Kumar A. Malaria in India: challenges and opportunities. J Biosci [Internet]. 2008 Nov;33(4):583–92. Available from: http://www.ncbi.nlm.nih.gov/pubmed/19208983

11. Shiv L, G.S S, P.K P. Status of Malaria in India. J Indian Acad Clin Med [Internet]. 5(1):19–23. Available from: http://medind.nic.in/jac/t00/i1/jact00i1p19.pdf

12. Sharma VP. Battling malaria iceberg incorporating strategic reforms in achieving Millennium Development Goals & malaria elimination in India. Indian J Med Res [Internet]. 2012 [cited 2014 Jun 3];136(December):907–25. Available from: http://icmr.nic.in/ijmr/2012/december/Centenary Review Article.pdf

13. Sharma VP, Mehrotra KN. Malaria resurgence in India: a critical study. Soc Sci Med [Internet]. 1986 Jan [cited 2014 Jul 15];22(8):835–45. Available from: http://www.ncbi.nlm.nih.gov/pubmed/3749959

14. Operational Manual for Implementation of Malaria Programme 2009 [Internet]. India: Directorate of National Vector Borne Disease Control Programme; 2009 p. 1–275. Available from: http://nvbdcp.gov.in/Doc/malaria-operational-manual-2009.pdf

15. Strategic Action Plan for Malaria Control in India [Internet]. India: Directorate of National Vector Borne Disease Control Programme; 2012 p. 1–114. Available from: http://nvbdcp.gov.in/Round-9/Annexure-2  Strategic action plan.pdf

16. NVBDCP | National Vector Borne Disease Control Programme [Internet]. [cited 2014 May 14]. Available from: http://nvbdcp.gov.in/malaria3.html

17. Bhatia R, Rastogi RM, Ortega L. Malaria successes and challenges in Asia. J Vector Borne Dis [Internet]. 2013 [cited 2014 May 15];50(December):239–47. Available from: http://www.searo.who.int/entity/world_health_day/2014/01-1434_rev.pdf

18. Odisha Health Sector Plan Policy and Activity Briefs [Internet]. 2012 [cited 2014 May 20]. p. 1–42. Available from: http://www.nrhmorissa.gov.in/writereaddata/Upload/Documents/OHSP.pdf

19. R Development Core Team (2011), R: A Language and Environment for Statistical Computing. Vienna, Austria : the R Foundation for Statistical Computing. [Internet]. [cited 2014 Jun 12]. Available from: http://www.r-project.org/.

20. Fox J. Time-Series Regression and Generalized Least Squares.Appendix to An R and S-PLUS Companion to Applied Regression [Internet]. 2002. p. 1–8. Available from: http://cran.r-project.org/doc/contrib/Fox-Companion/appendix-timeseries-regression.pdf

21. Dayer MJ, Jones S, Prendergast B, Baddour LM, Lockhart PB, Thornhill MH. Incidence of infective endocarditis in England, 2000–13: a secular trend, interrupted time-series analysis. Lancet [Internet]. Elsevier; 2014 Nov 18 [cited 2014 Nov 19]; Available from: http://www.thelancet.com/article/S0140673614620079/fulltext

22. Taljaard M, McKenzie JE, Ramsay CR, Grimshaw JM. The use of segmented regression in analysing interrupted time series studies: an example in pre-hospital ambulance care. Implement Sci [Internet]. 2014 Jan [cited 2014 Dec 5];9(1):77. Available from: http://www.pubmedcentral.nih.gov/articlerender.fcgi?artid=4068621&tool=pmcentrez&rendertype=abstract

23. Wagner AK, Soumerai SB, Zhang F, Ross-degnan D. Segmented regression analysis of interrupted time series studies in medication use research. J Clin Pharm Ther [Internet]. 2002;27:299–309. Available from: http://isites.harvard.edu/fs/docs/icb.topic79832.files/L06_Program_Evaluation_2/Segmented_Regression.Wagner.2002.pdf

24. Aregawi M, Lynch M, Bekele W, Kebede H, Jima D, Taffese HS, et al. Time Series Analysis of Trends in Malaria Cases and Deaths at Hospitals and the Effect of Antimalarial Interventions, 2001-2011, Ethiopia. PLoS One [Internet]. 2014 Jan [cited 2014 Nov 19];9(11):e106359. Available from: http://www.ncbi.nlm.nih.gov/pubmed/25406083

25. HINKLEY D V. Inference about the change-point in a sequence of random variables. Biometrika [Internet]. 1970 Apr 1 [cited 2014 Dec 19];57(1):1–17. Available from: http://biomet.oxfordjournals.org/content/57/1/1.abstract

26. Graneheim UH, Lundman B. Qualitative content analysis in nursing research: concepts, procedures and measures to achieve trustworthiness. Nurse Educ Today [Internet]. Elsevier; 2004 Feb 2 [cited 2014 Jul 9];24(2):105–12. Available from: http://www.nurseeducationtoday.com/article/S0260691703001515/fulltext

27. Valadez JJ, Devkota B, Pradhan MM, Meherda P, Sonal GS, Dhariwal A, et al. Improving malaria treatment and prevention in India by aiding district managers to manage their programmes with local information: a trial assessing the impact of Lot Quality Assurance Sampling on programme outcomes. Trop Med Int Health [Internet]. 2014 Jul 21 [cited 2014 Aug 12]; Available from: http://www.ncbi.nlm.nih.gov/pubmed/25039710

28. Anvikar A, Arora U, Das B, Dash A., Dhillon GP., Dua V., et al. Guidelines for Diagnosis and Treatment of Malaria in India [Internet]. India: NVBDCP,Government of India; 2009 p. 1–18. Available from: http://nvbdcp.gov.in/Doc/Guidelines_for_Diagnosis___Treatment.pdf

29. Shah NK, Kumar A, Valecha N. New global estimates of malaria deaths. Lancet [Internet]. 2012 Aug 11 [cited 2014 Mar 29];380(9841):560. Available from: http://www.thelancet.com/journals/a/article/PIIS0140-6736(12)61322-1/fulltext

30. Shah NK, Dhariwal AC, Sonal GS, Gunasekar A, Dye C, Cibulskis R. Malaria-attributed death rates in India. Lancet [Internet]. 2010 Mar 19 [cited 2014 May 15];377(9770):991. Available from: http://www.thelancet.com/journals/a/article/PIIS0140-6736(11)60378-4/fulltext

# Supporting information

**S1 Table.** **Status of Annual Malaria Incidence in 2003 (Baseline year), Odisha.**

**S1 Fig. Average seasonal variability of malaria incidence (2003–2013), Odisha.**
