# Supplementary material for: Trends in Malaria in Odisha, India—An Analysis of the 2003–2013 Time-Series Data from the National Vector Borne Disease Control Program
Source: PLoS One. 2016 Feb 11;11(2):e0149126. doi: 10.1371/journal.pone.0149126 (PMC4750863; doi:10.1371/journal.pone.0149126)
Supplement: S1 Table — (PDF) [file pone.0149126.s004.pdf]

	Name of the Districts with Annual Malaria Incidence < 2 (LOW)
1	Balasore
2	Bhadrak
3	Kendrapara
4	Jagatsingpur
5	Puri
	Name of the Districts with Annual Malaria Incidence 2 to < 5 (MODERATE)
6	Cuttack
7	Nayagarh
8	Baragarh
	Name of the Districts with Annual Malaria Incidence 5 to < 10 (HIGH)
9	Jajpur
10	Khurda
11	Ganjam
12	Jharsuguda
	Name of the Districts with Annual Malaria Incidence 10 or more (VERY HIGH)
13	Mayurbhanj
14	Keonjhar
15	Dhenkanal
16	Angul
17	Deogarh
18	Sundargarh
19	Sambalpur
20	Sonepur
21	Boudh
22	Kandhamal
23	Bolangir
24	Kalahandi
25	Rayagada
26	Koraput
27	Gajapati
28	Nuapada
29	Nawarangpur
30	Malkangiri
